# Using Statistical Time-Series Forecasting to Predict the Resting Heart Rate From Wearable Device Data: Case Report

**DOI:** 10.2196/91216

**Published:** 2026-09-25

**Authors:** Ying-Ju Chen, Chia-Yu Liu, Ya-Han Chang, Huh-Tswen Eric Lin, Tsang-Hai Huang

**Affiliations:** 1 Institute of Physical Education, Health and Leisure Studies National Cheng Kung University Tainan Taiwan; 2 Cycling Innovation Center Giant Manufacturing Co., Ltd. Taichung Taiwan; 3 Power Delivery Data Analytics Innovation Georgia Power (United States) Atlanta, GA United States

**Keywords:** heart rate, time-series forecasting, running, fatigue, wearables, case report

## Abstract

**Background:**

For endurance athletes, resting heart rate (RHR) is a well-known indicator of training load, physiological status, and readiness for upcoming training. Predicting the following day’s RHR would enable athletes and coaches to optimize training plans and make timely load adjustments.

**Objective:**

The aim of this study was to develop a statistical time-series forecasting model for RHR.

**Methods:**

Daily heart rate (HR) data (624 valid observations) collected from the personal wearable device of a single endurance runner (n=1) were used to establish a statistical time-series RHR forecasting model. This model was built using an autoregressive integrated moving average (ARIMA) model from the *sktime* package. The research framework evaluates wearable RHR forecasting models across 3 distinct phases. Phase 1 compared a naive persistence baseline against 4 dynamic seasonal autoregressive integrated moving average (SARIMA) models (using 1-day and 7-day rolling forecasts, with and without exogenous features) on a 75-25 train-test split. Phase 2 conducted a leave-one-feature-out ablation analysis on the top-performing model from phase 1. Finally, phase 3 assessed real-world “cold-start” viability by training a streamlined SARIMA model on the first 21 days of data.

**Results:**

In phase 1, the baseline naive forecaster yielded a mean absolute error (MAE) of 2.404. The SARIMA model using a 1-day rolling forecast with exogenous features and a chronological 75-25 split achieved an MAE of 1.712. An ablation analysis in phase 2 revealed that previous-day RHR and trimmed average active HR were the primary predictive drivers. Consequently, in phase 3, the streamlined SARIMA model, trained on just 21 days of initial data and using only the previous-day RHR and trimmed average active HR as 2 training features, demonstrated performance comparable to that of the full-history model.

**Conclusions:**

In the current case study, we preliminarily verified that a continuously updated SARIMA model trained on sufficient features can forecast future RHR in a single runner. Further study with a larger number of participants and the inclusion of more exogenous features will be needed to verify the framework’s applicability.

## Introduction

Regular exercise is considered an effective way to promote health and quality of life, and aerobic exercise has been shown to be an ideal option for improving and maintaining health [1]. Among various types of aerobic exercise, distance running has become one of the most popular [2,3]. However, although distance running significantly benefits overall health, excessive training can increase the risk of exercise-related injuries [4,5], particularly among recreational runners [6,7]. Overtraining syndrome, a major driver of exercise-related injuries, is a state of chronic fatigue and underperformance due to insufficient rest or recovery [8]. Crucially, this condition can be identified using heart rate (HR) data [9,10].

Previous studies have evaluated fatigue using physiological measurements (eg, HR, oxygen consumption, and lactic acid), calculated indices (eg, training impulse calculations based on HR and period [11]), and mathematical models that quantify the relationship between training and performance (eg, fitness-fatigue modeling [11] and influence curves [12]). These studies have established a large knowledge base [13-15], and many modern coaches design effective workouts based on these methodologies. However, recreational runners have been reported to rely on self-management rather than consult coaches or exercise training experts [16,17]. Thus, developing a self-monitoring method based on intuitive indicators would benefit recreational runners. With rapid developments in wearable technology, it is becoming feasible to continuously collect personal exercise and physiological data (eg, HR) that can be used to develop a self-monitoring algorithm [18].

The value of the wearable technology market reached more than US $100 billion [19], and global shipments exceeded 200 million units per year [20]. The market size was predicted to reach $380 billion by 2028 [21]. According to the American College of Sports Medicine Fitness Trends rankings [19,22-29], wearable technology has been ranked the top trend in the fitness industry since 2015. With the Industry 4.0 revolution, the integration of multiple sensors and chips into relatively small, portable devices is advancing rapidly [30]. Related technologies make it easy to accumulate large amounts of personal data. This includes, but is not limited to, measuring HR with photoplethysmography, recording exercise duration using a microprocessor master clock, collecting step (length, rate, and count) and sleep data with inertial measurement units (IMUs), and calculating total distance with GPS [31]. Mobile apps can now use commercially available algorithms to turn this collected data into a variety of information for users of these devices.

Measurements of HR variability (HRV) and resting HR (RHR) are 2 noninvasive methods for monitoring fatigue, and both can be measured with various commercial wearable devices. Of the two, measuring RHR is easier, and an elevated RHR can indicate overtraining. Manually measuring RHR or checking it on a smartwatch in the morning, right after waking up, is typically used to collect RHR as an indicator of overtraining [32-34]. By adding IMU data that detect users’ physical activity status, the continual collection of large-scale personal RHR data becomes feasible. This all-day RHR would be more personalized and could be a more potent indicator of physiological status and readiness for upcoming training. Moreover, as HR is a well-established parameter for calculating training load and defining training intensity [13-15], it is more intuitive and straightforward for recreational athletes to use RHR to monitor training status. Thus, if a full day’s RHR can be predicted in advance, it can open a window to make an early judgment or adjustments to upcoming training plans.

In previous studies, an autoregressive integrated moving average (ARIMA) model was applied to analyze HR in veterans with posttraumatic stress disorder and to verify the existence of distinguishable HR patterns and characteristics during hyperarousal events of posttraumatic stress disorder [35]. Besides, the ARIMA model has been shown to predict HR in young healthy male individuals up to 1 hour in advance [36]. The ARIMA model aims to detect autocorrelations in the data while accounting for the influence of time. Furthermore, the seasonal autoregressive integrated moving average (SARIMA) algorithm addresses the limitations of ARIMA by incorporating seasonal terms [37]. Thus, further prediction of a full day’s RHR by adopting the SARIMA model would be more valuable for exercise training.

The purpose of this study was to evaluate the performance of a SARIMA algorithm–based method in predicting the upcoming day’s RHR for an amateur runner.

## Methods

### Participant

A male amateur distance runner volunteered to participate in the current study. The male runner (aged 44 years, height=169.5 cm, weight=63.8 kg), who is a member of a local distance running club in southern Taiwan, has been participating in regular distance running for >5 years with a training frequency of 3 to 5 times per week and a running distance of >50 km per week. The runner’s personal best in a marathon is 3 hours and 4 minutes. To collect whole-day HR data, the participant was requested to wear a smartwatch (Forerunner 935, Garmin Ltd) throughout the experimental period, except during bathing and/or battery recharging. HR data were acquired via the smartwatch’s photoplethysmography sensor over 24 months.

### Data Acquisition

The data acquisition period spanned from July 2, 2019, to July 1, 2021 (731 days in total), and the personal smartwatch data were collected and synchronized to cloud storage via a commercial app (Garmin Connect Mobile, Garmin Ltd). Through the Garmin Health API, we further synchronized the personal data to our online database built with phpMyAdmin. According to the participant’s Garmin Connect Mobile record, 666 days were recorded as observation days. After consulting with the participant, the 65 missing observation days were mainly due to the participant not wearing the smartwatch for an entire day.

The device-generated default parameters included in the current study were whole-day HR and whole-day steps, which are automatically sampled every 15 seconds. By combining raw daily HR and step data, HR can be categorized into 2 distinct states: sleeping HR and active (nonsleeping) HR. We further calculated average sleep HR, minimum sleeping HR, maximum sleeping HR, active HR, minimum active HR, and maximum active HR (refer to operational definitions in Table 1). Because amateur athletes tend to train more on the weekends [38], Saturday and Sunday were designated as holidays and categorized as exogenous features in the present study. The operational definitions of each parameter are listed in Table 1. For the SARIMA model used in the current study, RHR was the only endogenous feature, while the remaining HR variables and the daily step count were exogenous features (Table 1). The periods for all parameters are shown in Figure 1. The workflow of the model operation is shown in Figure 2.

The parameters belonging to “Today” and “Yesterday” were used for training various models with the goal of predicting the RHR of the “Next day.”

As time progresses, previously accumulated data are continuously incorporated into the training dataset, enabling the recursive execution of predictions to reflect the most up-to-date information and maintain model accuracy over time.

**Table 1:** Operational definitions of parameters derived from the smartwatch.

Features	Operational definitions
Resting HR^a,b,c^	Average HR at rest during a single day
Average HR^c^	Average of HR values captured during the last 7 days
Maximum HR^c^	Maximum HR captured during a single day
Minimum HR^c^	Minimum HR captured during a single day
Average sleep HR	Average of HR values captured during a single continuous sleep period
Trimmed average sleep HR	Average of HR values captured during sleep after outliers were removed. This value is calculated using the *trim_mean* function from the *SciPy* library; it reduces the influence of extreme values and potential measurement errors by excluding the highest and lowest 25% of readings from the calculation.
Maximum sleep HR	Maximum HR captured during a single sleep period
Minimum sleep HR	Minimum HR captured during a single sleep period
Average active HR	Average of HR values captured during the nonsleeping period on a single day
Trimmed average active HR	Average of HR values captured during the nonsleeping period after outliers were removed. This value was calculated using the *trim_mean* function from the *SciPy* library; it reduces the influence of extreme values and potential measurement errors by excluding the highest and lowest 25% of readings from the calculation.
Maximum active HR	Maximum HR captured during the nonsleeping period on a single day
Minimum active HR	Minimum HR captured during the nonsleeping period on a single day
Steps^c^	Number of steps recorded during the monitoring period on a single day
Holiday	Saturday and Sunday were categorized as holidays.

^a^HR: heart rate.

^b^Endogenous features; data originally derived from the wearable device.

^c^Data derived from initial functions of the watch.

**Figure 1:**
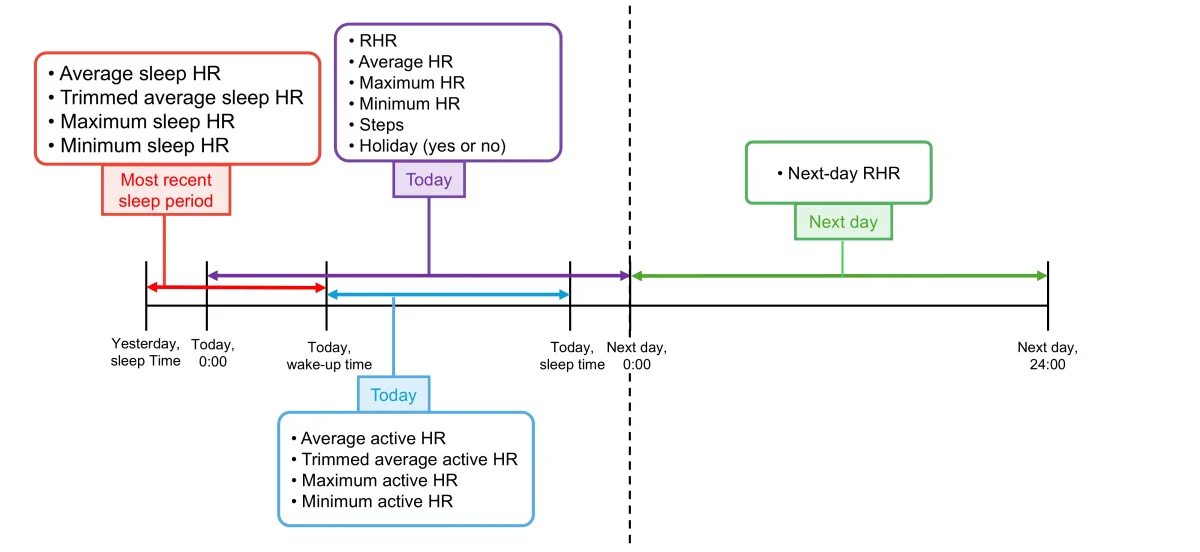
Periods of various parameters used in the study. HR: heart rate; RHR: resting heart rate.

**Figure 2:**
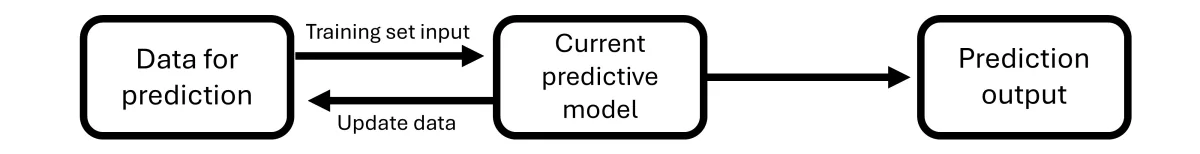
The workflow of the model operation.

### Data Partitioning

To prevent data leakage, the first 75% of the chronological timeline was used as the training set for initial model estimation, with the remaining 25% serving as the holdout testing set for rolling forecast evaluation.

### Data Cleaning

Raw data generated by smartwatches occasionally contain sensor artifacts or periods of nonwear. In the 666 observed days of this study, physiological readings of 0, which were mainly due to nonwear during the sleep period or a daytime gap in HR tracking, were identified as measurement anomalies and targeted for removal. Specifically, zero values were detected in the main features (RHR; n=14), active HR (n=30), and daily step count (n=1). Because several of these anomalies occurred concurrently on the same calendar day, a total of 42 daily observations were further removed from the 666 observed days. After these anomalous records were removed, the final cleaned dataset for modeling comprised 624 valid daily observations over the 24-month period.

A common challenge in preprocessing wearable data is handling the subsequent chronological gaps caused by these removals. To preserve the integrity of biological data, we explicitly chose not to synthesize or impute missing physiological data (eg, via linear interpolation or mean substitution), as mathematically inventing values during nonwear periods risks introducing artificial trends. Thus, these short chronological gaps were left unaltered. The *sktime* 0.40.1 (a Python library; Python Software Foundation 3.10.14) algorithmic architecture dynamically accommodates these naturally occurring missing intervals during forecasting, ensuring that the model trains exclusively on authentic physiological measurements.

### Algorithm and Modeling Process

#### Overview

The estimator used for modeling was the automated version of ARIMA (AutoARIMA) implemented in the *StatsForecast* package. To capture daily dynamics and weekly microcycles, the *StatsForecast* AutoARIMA model was implemented using the Hyndman-Khandakar algorithm for automated hyperparameter selection, minimizing the corrected Akaike information criterion. Exogenous features were integrated directly as regressors within a SARIMA framework.

To evaluate the predictive performance and practical viability of the models for wearable devices, our experimental design was structured into 3 phases.

#### Phase 1: Full-History Model

The dataset was partitioned chronologically into a 75% training set and a 25% testing set. A naive forecaster (which predicted the next day’s RHR as the current day’s RHR) was implemented as a baseline model to prove the effectiveness of the statistical forecasting approach over simple persistence. We then evaluated 4 dynamically updated SARIMA models (Figure 3A and 3B): 1-day rolling forecast with features, 1-day rolling forecast without features, 7-day rolling forecast with features, and 7-day rolling forecast without features. The models were initially fitted to the 75% training data, and a rolling update strategy was used to iterate over the 25% test data. The information for the automated hyperparameter selection setup, ARIMA(*p,d,q*)(*P,D,Q*)*_m_*, is provided, in which *p* and *P* represent nonseasonal and seasonal autoregressive orders, respectively; *d* and *D* represent nonseasonal and seasonal differencing orders, respectively; *q* and *Q* represent nonseasonal and seasonal moving average orders, respectively; and *m* represents the seasonal period (days).

**Figure 3:**
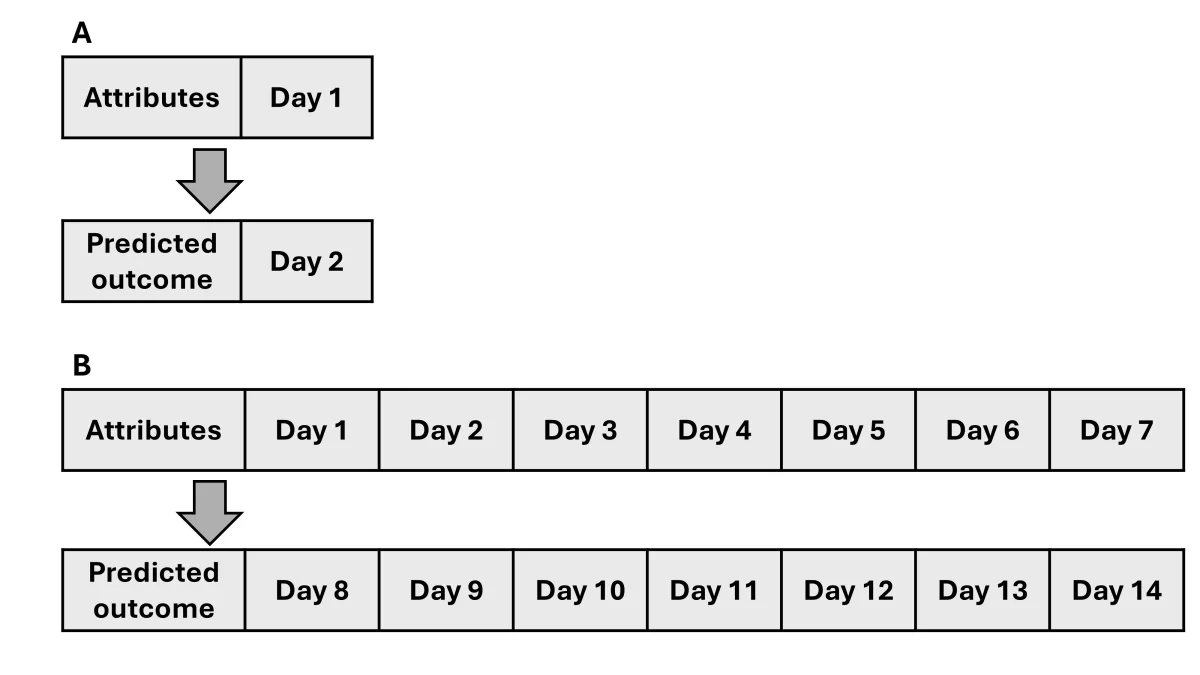
The 1-day and 7-day rolling update strategies. The automated version of the autoregressive integrated moving average (AutoARIMA) model from the sktime package is trained using attributes (A) from day 1 to predict the outcome on day 2; and (B) from days 1 to 7 to predict outcomes from days 8 to 14.

#### Phase 2: Ablation Analysis

To isolate the predictive contribution of the exogenous features and optimize computational efficiency, a leave-one-feature-out (LOFO) ablation analysis was conducted on the highest-performing model from phase 1, which was the 1-day rolling forecast with features, measured by mean absolute error (MAE). Each feature was systematically omitted, and the model was retrained and evaluated. Features that increased MAE were retained, while features that degraded performance due to multicollinearity were discarded.

#### Phase 3: Short-Initiation Model

To test the commercial viability of deploying the algorithm to a new user without requiring months of historical data, we simulated a “Cold-Start” environment. Using only the 2 features identified in phase 2, a streamlined SARIMA model was trained on the first 21 days of the dataset. The model then used a 1-day rolling update strategy across the remaining 603 days of streaming data, updating its internal state daily to adapt to the athlete’s nonstationary training cycles.

Metrics of MAE (measured in bpm), mean absolute percentage error (MAPE; measured in percentage), mean squared error (MSE; measured in beats^2^/min^2^), root mean square error (RMSE; measured in bpm), and scatter index (SI=RMSE/average RHR) were used to evaluate model performance across all phases.

### Ethical Considerations

The study was approved by the National Cheng Kung University Institutional Review Board (A-ER-106-493), and all procedures were conducted in accordance with the ethical standards of the Declaration of Helsinki. The participant signed a written informed consent form and agreed to wear a smartwatch (Forerunner 935) throughout the experimental period. The participant was informed that participation was voluntary and that he had the right to withdraw at any time. All data generated by the smartwatch, including HR metrics synchronized from the Garmin Health database, were deidentified to ensure participant confidentiality. Upon completion of the data acquisition period, the participant was provided with the smartwatch as compensation for his involvement in this case study.

## Results

### Overview

In Table 2, descriptive statistics for each parameter are listed. During the observation period, the distance runner demonstrated a typical pattern of RHR (mean 49.099, SD 2.392 bpm) and sleeping HR (mean 50.952, SD 2.495 bpm). The average daily steps (mean 19,680, SD 10,277 steps/day) also indicated a regular exercise training behavior in the current participant.

**Table 2:** Descriptive statistics of endogenous and exogenous features.

Models	Parameters, mean (SD)
Resting HR^a,b,c^	49.099 (2.392)
Average HR^c^	75.313 (5.060)
Minimum HR^c^	42.103 (3.501)
Maximum HR^c^	157.471 (14.452)
Average sleep HR	50.925 (2.495)
Trimmed average sleep HR	50.833 (2.573)
Maximum sleep HR	76.213 (9.859)
Minimum sleep HR	43.431 (3.325)
Average active HR	82.974 (6.539)
Trimmed average active HR	78.589 (6.598)
Maximum active HR	157.471 (14.452)
Minimum active HR	45.197 (4.364)
Steps^c^	19,680 (10,277)

^a^HR: heart rate.

^b^Endogenous features; data originally derived from the wearable device.

^c^Data derived from initial functions of the watch.

### Phase 1: Full-History Model

In phase 1, five models were trained with the chronologically split dataset (75% training and 25% testing). To establish a comparative baseline, a naive forecaster (which predicted the next day’s RHR as the current day’s RHR) was implemented, yielding an MAE of 2.404 bpm. The remaining 4 models used a SARIMA framework, evaluating 1-day and 7-day rolling forecasting horizons, both with and without exogenous features.

The model performance metrics and automatically selected hyperparameter orders are detailed in Table 3. The models trained with exogenous features demonstrated significantly higher predictive accuracy (Figures 4A and B). The optimal configuration was the 1-day rolling forecast with features, which achieved an MAE of 1.712 bpm and an RMSE of 2.315 bpm, representing a nearly 30% error reduction compared to the naive baseline. The automated hyperparameter selection for this optimal model (Table 3) revealed an ARIMA(0,0,0)(2,0,0)_7_ structure; the exogenous features successfully explained day-to-day variance (Table 4), while the seasonal autoregressive terms captured the athlete’s weekly training cycle.

Conversely, models trained without features were relatively less effective in predicting the upcoming daily RHR. In addition to the relatively higher MAE of the nonfeature models (Table 3), the statistical variance metrics (Table 4) and graphs (Figures 4C and D) also showed that the nonfeature models underfit the data.

**Table 3:** Model performance metrics for the training set.

Models		MAE^a^ (bpm)	MAPE^b^ (%)	MSE^c^ (beats^2^/min^2^)	RMSE^d^ (bpm)	SI^e^	Order^f^
Baseline: naive forecast		2.404	4.8	9.981	3.159	0.064	—^g^
**Trained with features**							
	7-day forecast	1.777	3.6	5.7	2.388	0.048	ARIMA(0,0,0)(2,0,0)_7_
	1-day forecast	1.712	3.5	5.361	2.315	0.047	ARIMA(0,0,0)(2,0,0)_7_
**Trained without features**							
	7-day forecast	1.988	4.0	6.601	2.569	0.052	ARIMA(1,1,1)(0,0,0)_1_
	1-day forecast	1.901	3.8	6.118	2.474	0.050	ARIMA(1,1,1)(0,0,0)_1_

^a^MAE: mean absolute error.

^b^MAPE: mean absolute percentage error.

^c^MSE: mean squared error.

^d^RMSE: root mean square error.

^e^SI: scatter index; SI=RMSE/average resting heart rate.

^f^The notation of the autoregressive integrated moving average (ARIMA) model with or without features is presented as ARIMA(p,d,q)(P,D,Q)m, where p and P represent the nonseasonal and seasonal autoregressive orders, respectively; d and D represent the nonseasonal and seasonal differencing orders, respectively; q and Q represent the nonseasonal and seasonal moving average orders, respectively; and m represents the seasonal period (days).

^g^The naive forecast does not include hyperparameter orders.

**Figure 4:**
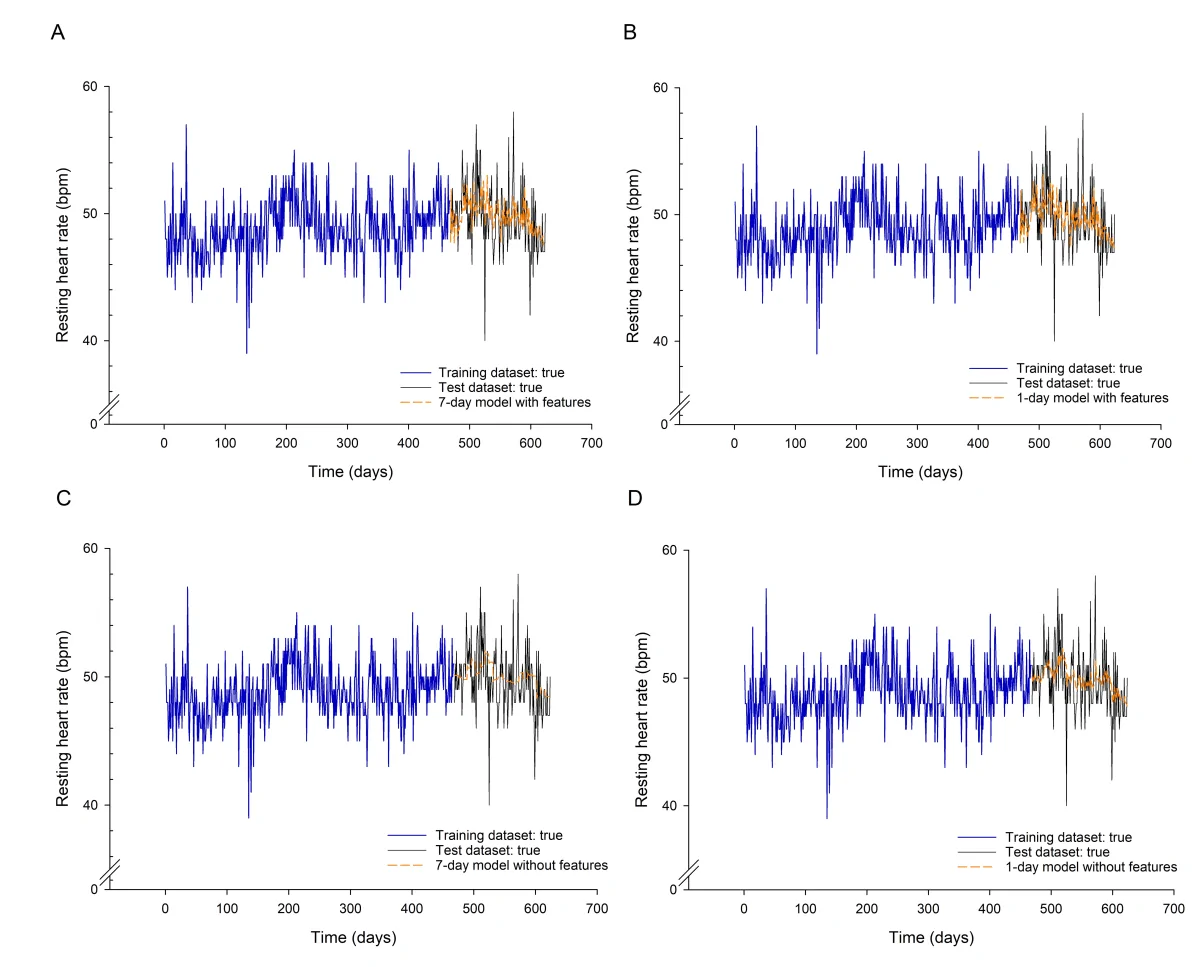
Performance of 4 trained models on the test dataset. (A) Seven-day trained model with features, (B) 1-day trained model with features, (C) 7-day trained model without features, and (D) 1-day trained model without features. The blue solid curve represents the original wearable device–measured resting heart rate (RHR) in the training dataset; the black solid curve represents the original wearable device–measured RHR in the test dataset; and the orange dashed curves represent the RHR predicted by the 4 models.

**Table 4:** Descriptive statistical values of observed and forecast resting heart rate (RHR) values (bpm) on test data.

Models			RHR, mean (SD)		RHR, variance
Observed test data			49.724 (2.584)		6.678
Baseline: naive forecast			49.731 (2.586)		6.688
**Trained with features**					
	7-day forecast	49.843 (1.148)		1.319	
	1-day forecast	49.731 (1.231)		1.514	
**Trained without features**					
	7-day forecast	50.035 (0.788)		0.622	
	1-day forecast	49.883 (0.900)		0.810	

### Phase 2: Ablation Analysis

To isolate the predictive value of individual features used in the optimal phase 1 model, a LOFO ablation analysis was conducted (Table 5). As RHR is a highly stable metric (typically fluctuating only 1-3 bpm daily), absolute error boundaries are inherently tight. However, the ablation revealed significant relative impacts. Omission of the previous day’s RHR was the primary driver of predictive degradation, resulting in a 7.5% relative increase in the MAPE (rising from 3.45% to 3.71%) and an MAE increase of 0.13 bpm. The trimmed mean active HR also contributed positively to the forecast.

Conversely, the simultaneous inclusion of the remaining 10 aggregated metrics (such as sleep HR and step count) exhibited a decrease in error when these features were removed. This confirms that highly complex, multivariable feature sets introduce multicollinearity and mild overfitting when predicting stable physiological baselines. Consequently, to maximize computational efficiency without sacrificing accuracy, a lean 2-feature (ie, RHR and trimmed average active HR) framework was implemented in the phase 3 simulation.

**Table 5:** Ablation analyses of features in the 1-day forecast model.

Dropped features	Ablated MAE^a^ (bpm)	MAE increase (bpm)	Ablated MAPE^b^ (%)	MAPE increase (%)
Resting HR^c^	1.842	0.130	3.710	0.260
Trimmed average active HR	1.728	0.017	3.486	0.036
Minimum HR	1.719	0.007	3.469	0.019
Maximum HR	1.714	0.002	3.460	0.010
Steps	1.712	0	3.455	0.005
Average active HR	1.712	0	3.451	0.001
Maximum sleep HR	1.709	−0.003	3.446	−0.004
Trimmed average sleep HR	1.709	−0.003	3.446	−0.004
Holiday	1.708	−0.004	3.443	−0.007
Average sleep HR	1.705	−0.007	3.439	−0.011
Minimum active HR	1.698	−0.013	3.425	−0.026
Average HR	1.691	−0.021	3.418	−0.032

^a^MAE: mean absolute error.

^b^MAPE: mean absolute percentage error.

^c^HR: heart rate.

### Phase 3: Short-Initiation Model

According to the ablation analyses, a feature set consisting exclusively of RHR and trimmed average active HR was established. A new 1-day rolling SARIMA model was established using only the first 21 days of the dataset. Over the subsequent 603-day period, this continuously updating model achieved an MAE of 1.63 bpm and an RMSE of 2.16 bpm (Table 6). As detailed in Table 6, this short-initiation model demonstrated comparable performance across the testing period.

**Table 6:** Comparison of full-history and short-initiation models.

Models	MAE^a^ (bpm)	MAPE^b^ (%)	RMSE^c^ (beat^2^/min^2^)	MSE^d^ (bpm)	SI^e^	Forecast mean (SD)	Forecast variance
Phase 1: full-history (all features)	1.712	3.5	2.315	5.361	0.047	49.731 (1.231)	1.514
Phase 3: short-initiation (2 features)	1.630	3.3	2.163	4.679	0.044	49.083 (1.263)	1.594

^a^MAE: mean absolute error.

^b^MAPE: mean absolute percentage error.

^c^MSE: mean squared error.

^d^RMSE: root mean square error.

^e^SI: scatter index; SI=RMSE/average resting heart rate.

## Discussion

### Principal Findings

In the present study, continuous time-series HR data from a commercial wearable device were used to forecast the next-day (1-day rolling forecast) or next-week (7-day rolling forecast) RHR for a distance runner. Within the scope of this case study, a 3-phase evaluation indicated that multivariate SARIMA models performed better than univariate approaches and naive baselines. These results suggest that predicted RHR derived from prior HR parameters may serve as a readiness reference for upcoming exercise training.

### Contribution of HR-Generated Features

In the current study, our phase 2 ablation analysis revealed that combining RHR with exogenous features improved accuracy. Conversely, including all features led to multicollinearity and mild overfitting. As the current study aimed to develop a prediction tool to support a healthy adult distance runner, exercise training was expected to be the primary contributor to fluctuations in daily HR data, as different levels of exercise training exert distinct influences on autonomic modulation, HR indices, and HRV [39-41]. In this case, using HR-derived features as data sources was streamlined and sufficient to create a statistically useful model. In the current study, we found that both 1-day and 7-day SARIMA models with exogenous features could generate RHR predictions that can be used to inform sports training practice. The 1-day model can serve a precautionary function, allowing the athlete to adjust training, while the 7-day RHR prediction can forecast readiness over the next week, helping the athlete plan the upcoming microcycle training schedule.

However, the current study shows that the true day-to-day RHR fluctuations of the runner ranged from 0 to 10 (bpm), whereas the 1-day and 7-day SARIMA models with exogenous features showed predicted RHR changes of only 0 to 4 (bpm) across calendar days. Theoretically, HR is easily affected by various internal or external environments [32]. A forthcoming full-day RHR is sensitive to various stressors [42-45] (eg, life events, diet, and clinical symptoms) that occur randomly and are currently out of scope for these exogenous feature-included SARIMA models. Some of these stressors can have longer-term effects on the next-day or next-week HR, such as influenza or seasonal allergies [44,46], while some are temporary, such as dehydration or caffeine intake, which can cause a heart rate shift [47,48]. In contrast to clinical symptoms, random stressors might merely induce a diurnal HR shift rather than serve as determining factors for the subsequent day’s HR. In other words, these factors are transient stressors that leave no residual effects for the next day. Taken together, the predictive capacity of the current exogenous feature-included SARIMA models may be compromised because they do not include other effective stressors beyond exercise training.

Nevertheless, this case study established a streamlined model for predicting future RHR that is applicable to exercise training practices. To develop a more accurate predictive model, additional endogenous or exogenous features need to be included and investigated.

### HR vs HRV

Technically, HRV indices, which are affected by the autonomic nervous system, can be used to indicate or evaluate the status of overtraining [49,50]. There are multiple HRV indices representing sympathetic and parasympathetic nerve activity, such as RR intervals plotted against time, the RR intervals’ standard deviation (SDNN), the root mean square successive difference (rMSSD), and the amount of neighboring RR intervals varying >50 milliseconds expressed in percentage (pNN50). However, despite their academic value, HRV analysis outputs are neither intuitive nor user-friendly. Moreover, the validity of HRV analyses is relatively sensor-dependent. In reality, comparing HRV measurements from commercial wearable devices indicates that the overall validity is currently poor under free-living conditions [51]. Therefore, using RHR is preferred as an indicator of long-term monitoring of training or physiological status due to its relative feasibility and reliability.

### Limitations

A primary limitation of statistical time-series forecasting in this context is its tendency to smooth extreme daily fluctuations, which likely accounts for the lower prediction variance observed relative to the ground-truth data. To better capture these sharp RHR dynamics, future studies should incorporate additional internal and external stressors as feature inputs and explore machine learning or deep learning architectures beyond traditional statistical models.

Additionally, missing data due to nonwear or improper device placement may pose a practical challenge for continuous RHR forecasting. Under the current ARIMA model, if yesterday’s data are unavailable, feature data from 2 days prior are substituted to predict today’s RHR. Within the phase 3 testing set, the MAE values for predicted RHR, with and without yesterday’s data feature, were 1.637 and 1.517 bpm, respectively. This indicates that nonequidistant sampling did not compromise forecast performance in this case report. However, because the participant of the current study demonstrated high compliance and maintained a consistent training schedule, his relatively stable physiological baseline may have mitigated the impact of missing data. In broader real-world applications, nonwear intervals may need to be modeled as an exogenous feature to account for underlying physiological shifts associated with life event–related disruptions, such as sudden illness or prolonged sedentary periods.

### Practical Applications

Predicting an athlete’s next-day physiological recovery metrics, such as RHR and HRV, is an evolving area in sports science and predictive analytics. As previously noted, existing literature using classical time-series modeling is limited; for instance, only one study has used an ARIMA model to predict the next 1-hour HR in healthy young male individuals from a 24-hour database [36]. In addition, a recent study demonstrated that machine learning approaches (ie, support vector regression [SVR] and Extreme Gradient Boosting [XGBoost]) could predict next-day HRV by using external training loads (total mechanical work in kilojoules) and internal training loads (rate of perceived exertion) as primary feature sources [52], which is somewhat complicated and dependent on multiple sensors. Thus, extending the forecasting horizon to predict next-day HR would provide a more intuitive and practically applicable framework for managing athletic training. In phase 3 of our analysis, we tested the potential commercial applicability of the current models. In contrast to the >400-day model training, phase 3 adopted a short-initiation model that included only a 21-day training period with 2 features (ie, RHR and the trimmed average active HR). The comparable outcomes between the full-history and short-initiation models suggest that a 3-week period of model training may be sufficient to establish a mathematical RHR predictive function applicable to exercise training practice.

As HR is a straightforward index readily available from commercial devices, predicted RHR can be used as an indicator of future physiological status. According to the present study’s findings, 1-day or 7-day RHR forecasts can serve as a reference for short-term adjustments or longer-term planning for upcoming training. It would be worthwhile to further apply this methodology to various issues in endurance training. In addition, as an abnormally raised RHR is also an indicator of up-regulated sympathetic activity [53], future RHR forecasts can be a health security solution to protect people from extremely high stress.

### Conclusions

In conclusion, using longitudinal HR measurements, we developed a statistical time-series framework for predicting the next-day and next-week RHR of a distance runner. By isolating the most predictive features, we demonstrated that 3 weeks of data can initiate a lean, continuously updating SARIMA model that achieves performance comparable to that of the full-history model. Further studies that include more exogenous features and a larger number of participants are needed to verify the framework’s applicability.

## Abbreviations

ARIMA: autoregressive integrated moving average
AutoARIMA: automated version of autoregressive integrated moving average
HR: heart rate
HRV: heart rate variability
IMU: inertial measurement unit
LOFO: leave-one-feature-out
MAE: mean absolute error
MAPE: mean absolute percentage error
MSE: mean squared error
pNN50: the amount of neighboring RR intervals varying >50 milliseconds expressed in percentage
RHR: resting heart rate
RMSE: root mean square error
rMSSD: root mean square successive difference
SARIMA: seasonal autoregressive integrated moving average
SDNN: the RR intervals’ standard deviation
SVR: support vector regression
SI: scatter index
XGBoost: Extreme Gradient Boosting

## Data Availability

The datasets generated or analyzed during this study are not publicly available due to privacy and ethical considerations, but deidentified data are available from the corresponding author on reasonable request.
